# Preparation of a Fluorocarbon Polymerizable Surfactant and Its Application in Emulsion Polymerization of Fluorine-Containing Acrylate

**DOI:** 10.3390/polym9110606

**Published:** 2017-11-14

**Authors:** Meng Zhao, Youhai Yu, Zhewen Han, Hui Li

**Affiliations:** 1 School of Materials Science and Engineering, East China University of Science and Technology, Shanghai 200237, China; happyzhaomeng513@163.com; 2 Center for Advanced Low-Dimension Materials, Donghua University, Shanghai 201620, China; yuyouhai@dhu.edu.cn

**Keywords:** fluorocarbon polymerizable emulsifier, emulsion polymerization, emulsifier, fluorinated polymer, characterization

## Abstract

A novel polymerizable fluorocarbon surfactant, perfluoro (4–methyl–3, 6–dioxaoct–7–ene) sodium sulfonate (PSVNa), was synthesized and characterized. The fluorocarbon surfactant PSVNa and its mixture PSVNa/SDS were used as emulsifiers during the emulsion polymerization of DFHMA/MMA. The investigation of polymerization kinetics, particle size, and stability of the emulsions revealed that PSVNa has excellent emulsifying properties. The NMR spectrum of the copolymer and the detection of residual PSVNa show that more than 95% of the fluorocarbon surfactants have been linked to the polymer chains by radical polymerization, which will greatly reduce the environmental pollution caused by fluorinated surfactants.

## 1. Introduction

Fluorinated polymers, with excellent environmental stability, low coefficient of friction water and oil repellency, biocompatibility, excellent thermal stability, chemical resistance, and low interfacial free energy, have been widely used in the defense and high-tech industries [1,2]. Most fluoropolymers, such as PTFE, PVDF, and fluorinated polyacrylate, are prepared by free radical polymerization in aqueous phases [3]. Fluorocarbon surfactants based on perfluorooctanoic acid (PFOA) and perfluorooctane sulfonate (PFOS) have been widely adopted in the conventional processes of fluoropolymers manufacture. However, since 2015, PFOA and PFOS have been forbidden in many countries due to their long-term environmental pollution. Therefore, it is urgent to develop novel environmentally friendly fluorocarbon surfactant to replace PFOA- and PFOS-based ones. Several kinds of fluorocarbon surfactants have been developed as substitutes of PFOA in recent years, and the main idea is to shorten the perfluorocarbon chains, or to replace perfluoroalkyl with fluorinated polyether segments to reduce their bioaccumulation. However, these small molecules, containing fluorinated segments, can still pollute the environment due to their excellent stability in nature environment [4,5,6,7].

Polymerizable surfactants, including cationic, anionic, nonionic, and zwitterionic polymerizable surfactants, can react with monomers in the presence of an initiator or at high temperatures, connect to the polymer chain in the form of covalent bonds, and become part of the polymer [4,8,9,10]. Therefore, it is possible to reduce the residue of surfactants to zero or a very small amount when it was used as an emulsifier during emulsion polymerization [11,12]. In recent years, several cationic fluorocarbon polymerizable surfactants have been prepared, and their surface activities have been investigated. However, the study of anionic fluorocarbon polymerizable surfactant has rarely been reported. Meanwhile, the polymerization activity of these surfactants has not been studied [13,14].

Here, a novel type of anionic fluorocarbon polymerizable surfactant, perfluoro(4–methyl–3, 6–dioxaoct–7–ene) sodium sulfonate (PSVNa) was prepared with an existing material in a very simple method. The structure and surface property of PSVNa have been characterized and its application as an emulsifier in emulsion polymerization of fluorinated acrylate was investigated. More importantly, the FTIR and NMR results of the polymer show that the surfactant is indeed covalently linked to the polymer chain, and the PSVNa residue test shows that more than 95% of the surfactants have been polymerized. The development of this kind of surfactant is beneficial to environmental protection due to the lower residue. Meanwhile, the introduction of appropriate hydrophilic groups may bring new properties and applications for the polymers.

## 2. Materials and Methods

### 2.1. Materials

Perfluoro (4–methyl–3, 6–dioxaoct–7–ene) sulfonyl fluoride (PSVE, 99%) and pentadecafluorooctanoic acid were obtained from Shanghai 3F New Material Co. Ltd. (Shanghai, China); methyl methacrylate (MMA, 99%), sodium hydroxide (NaOH), ammonia water (25%), acetone, potassium persulfate (KPS), and sodium dodecyl sulfate (SDS) were purchased from Shanghai Lingfeng Chemical Co. Ltd. (Shanghai, China); dodecafluoroheptyl methacrylate CH_2_=C(CH_3_)COOCH_2_CF(CF_3_)CFHCF(CF_3_)_2_ (DFHMA, 98%) was obtained from XEOGIA Fluorine-Silicon Chemical Co. Ltd. (Harbin, China), The inhibitors in monomers were removed before use by alumina column. Ammonium perfluorooctanoate (PFOA) was synthesized with pentadecafluorooctanoic acid and ammonia water as raw materials in our lab.

### 2.2. Preparation of Perfluoro(4–methyl–3, 6–dioxaoct–7–ene) Sodium Sulfonate (PSVNa)

**Scheme 1:**
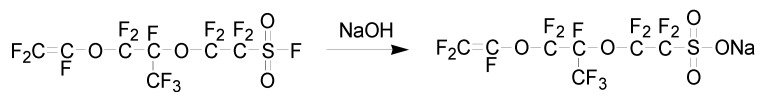
Synthesis of perfluoro(4–methyl–3, 6–dioxaoct–7–ene) sodium sulfonate.

The compound was synthesized according to Scheme 1. Perfluoro(4–methyl–3, 6–dioxaoct–7–ene) sulfonyl fluoride (22.3 g, 0.05 mol) was dissolved in 50 mL of acetone in a round bottom flask; 10% NaOH solution was added slowly into the flask till the pH value reached 8–9. The solvent was removed under high vacuum, and the solid portion was dissolved in acetone. Then the mixture was filtered and the solvent (acetone) was removed under high vacuum at 40 °C. Perfluoro (4–methyl–3, 6–dioxaoct–7–ene) sodium sulfonate was obtained with 87% yield.

### 2.3. Preparation of DFHMA/MMA Copolymer by Emulsion Polymerization

**Scheme 2:**
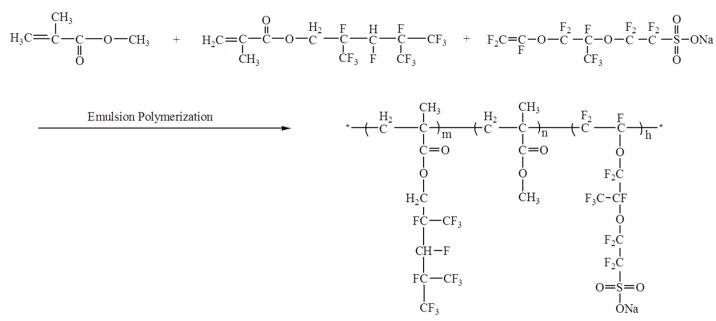
Process of emulsion polymerization.

Polymerization (Scheme 2) was carried out in a four-necked, round bottom flask fitted with a reflux condenser, a stirrer, a thermometer, and an addition funnel with a nitrogen gas inlet. A series of fluorinated acrylate hybrid dispersions were prepared by the copolymerization of DFHMA (40%) and MMA (60%) in the presence of PSVNa or SDS/PSVNa (2%) as an emulsifier. In the meantime, the same emulsion polymerization with ammonium perfluorooctanoate (APFO) as an emulsifier was carried out for comparison. The emulsion polymerization was carried out with KPS/NaHSO_3_ (0.4%) as an initiator at 55–60 °C for 4 h.

### 2.4. Characterization

#### 2.4.1. FTIR Analysis

Fourier transform infrared (FTIR) spectra were acquired with a MAGNA-IR550 Fourier transform infrared spectrometer (Nicolet Instrument Co., Madison, WI, USA). For each sample, 32 scans at a 4 cm^−1^ resolution were collected in absorption group mode.

#### 2.4.2. 19F NMR Analysis

^19^F NMR spectra were recorded on Bruker AVANCE III NMR spectrometers operating at 500 MHz. D_2_O and CDCl_3_ were used as solvents for PSVNa and the copolymers, respectively.

#### 2.4.3. Thermo Gravimetric Analysis (TGA)

The samples were measured with a DuPont 1090B thermo gravimetric analyzer (TA Instruments, New Castle, DE, USA) with a heating rate at 10 °C/min from ambient temperature to 500 °C under a nitrogen atmosphere.

#### 2.4.4. Differential Scanning Calorimetry (DSC)

DSC analysis was carried out with a Q2000 different scanning calorimeter instrument (TA Instruments, New Castle, DE, USA). Data were collected at 10 °C/min from 40 to 250 °C.

#### 2.4.5. Surface Tension Measurements

Surface tension was measured using a QBZY-1 (Fangrui Instrument Co., Shanghai, China) inter-facial tensionmeter employing platinum piece at 25.0 ± 0.1 °C. The tensionmeter was calibrated using triple distilled water. The surfactant solutions were aged for 12 h prior to the measurements. For the measurements, an adequate amount of concentrated surfactant solution was added into 20 mL of water in order to change the surfactant concentration from concentrations well below the critical micelle concentration (CMC) to at least 2–3 times the CMC.

#### 2.4.6. Particle Size and Distribution of Emulsion

The particle size and particle size distributions of the emulsions were measured with photon correlation spectroscopy on a Malvern Zetasizer 3000 HS (Malvern Instruments Ltd., Malvern, UK).

#### 2.4.7. Determination of Conversion Rate

The conversion was measured by gravimetric analysis. One to two milliliters of emulsion was cast onto a culture vessel with two drops of inhibitor and dried at 200 °C until a constant weight was attained. The final conversion rate was calculated by the following equation:

(1) $$\mathrm{conversion}\;(\mathrm{wt}\;\%)=\frac{(\frac{W_{1}-W_{0}}{W_{2}-W_{0}})\times W_{3}-W_{4}}{W_{5}}\times 100\%$$

*W*_0_ is the weight of the culture dish. *W*_1_ and *W*_2_ are the weight of latex before and after drying to the constant weight, respectively; *W*_3_ is the total mass of all the materials; *W*_4_ is the weight of all the materials except water and monomers in the culture dish; *W*_5_ is the total weight of monomers.

The sample was taken out every 2 min in the first half hour and every 10 min after.

#### 2.4.8. Detection of PSVNa Residue in Latex Films

Ten grams of latex films with only PSVNa as an emulsifier were cut into pieces and put into a Soxhlet extractor to extract 12 h by the mixture of water and methanol (200 mL), and the resulting solution was diluted to different concentrations. LC-MS was used to detect the signal of PSVNa in diluted solution. At the same time, different concentrations of standard solution of PSVNa including 500, 100, 50, 20, 10, 5, and 1 ppm were also detected by LC-MS. LC-MS was tested by XEVO TQD from Waters Co. (Milford, MA, USA).

#### 2.4.9. Immersion Behavior of the Synthesized Latex Films

Contact angle measurements were carried out with a JC-2000A contact angle goniometer (ZhongChen Co., Shanghai, China) at room temperature (20 °C) and the results reported are the mean values of 5 replicates.

## 3. Results and Discussion

### 3.1. Characterization of PSVNa

**Figure 1:**
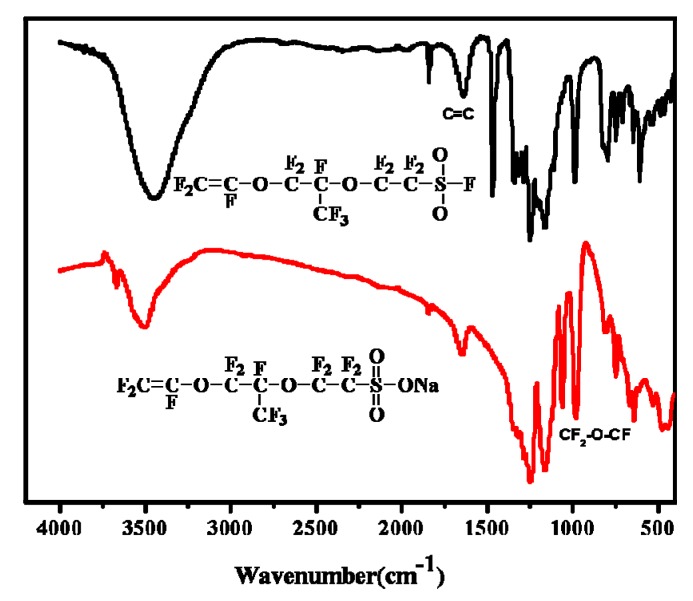
FTIR spectra of perfluoro (4–methyl–3, 6–dioxaoct–7–ene) sulfonyl fluoride (PSVE) and perfluoro (4–methyl–3, 6–dioxaoct–7–ene) sodium sulfonate (PSVNa).

PSVNa was prepared by a very simple procedure from PSVE and the product was characterized. Figure 1 demonstrates the typical FTIR spectra of the original material PSVE and the final product PSVNa. In the spectra of PSVE, the strong characteristic stretching absorption bands of –CF_2_– and –CF_3_ are at 1100–1300 cm^−1^ and the strong characteristic absorption peak of CF_2_–O–CF is at 989 cm^−1^. The absorption peak at 1650 cm^−1^ were the characteristic band of C=C and the spectra of O=S=O appears at 1000–1100 cm^−1^. In the spectra of PSVNa, the characteristic stretching absorption peaks of CF_2_–O–CF, O=S=O, –CF_2_– and –CF_3_ also appear with slight shifts. This indicates that the PSVNa is successfully synthesized.

**Figure 2:**
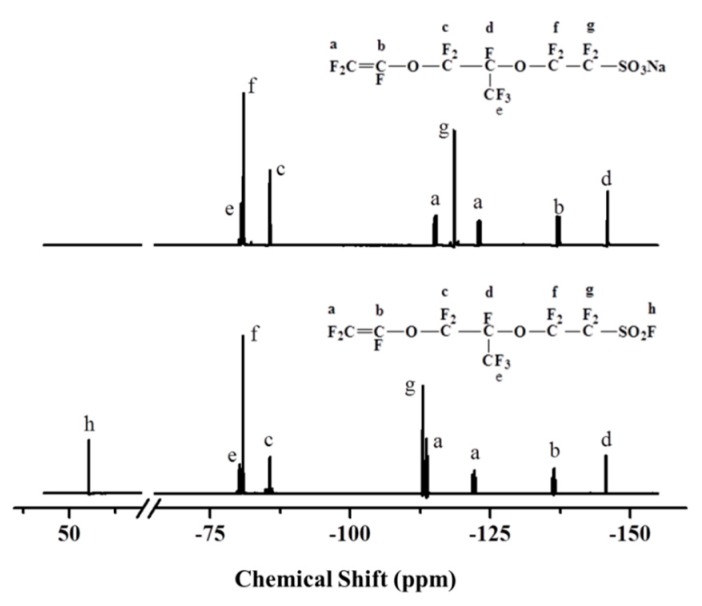
^19^F NMR spectrum of PSVE and PSVNa.

Figure 2 shows the ^19^F NMR spectrums of PSVE and PSVNa. All the peaks are well assigned in this figure. Peak h disappeared because the –SO_2_F transferred to –SO_3_Na, and the signal of Peak g exhibited a slightly downfield shift from δ = −118 ppm in PSVE to δ = −113 ppm in PSVNa.

**Figure 3:**
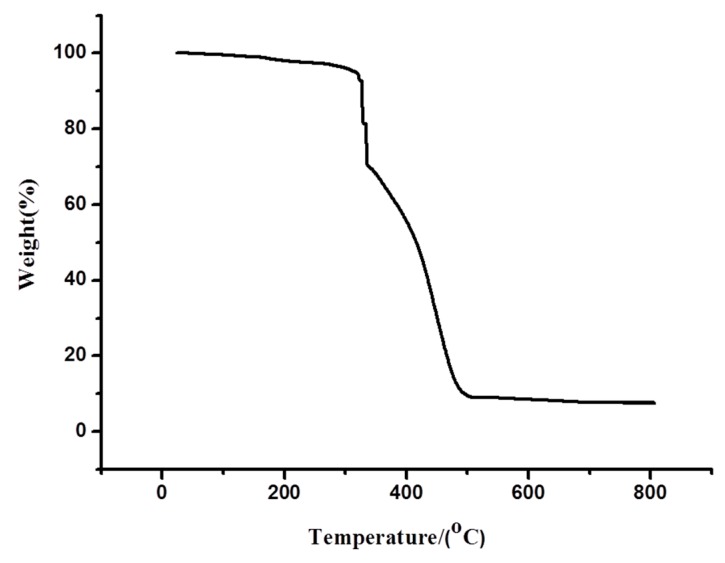
TGA curves of PSVNa.

**Figure 4:**
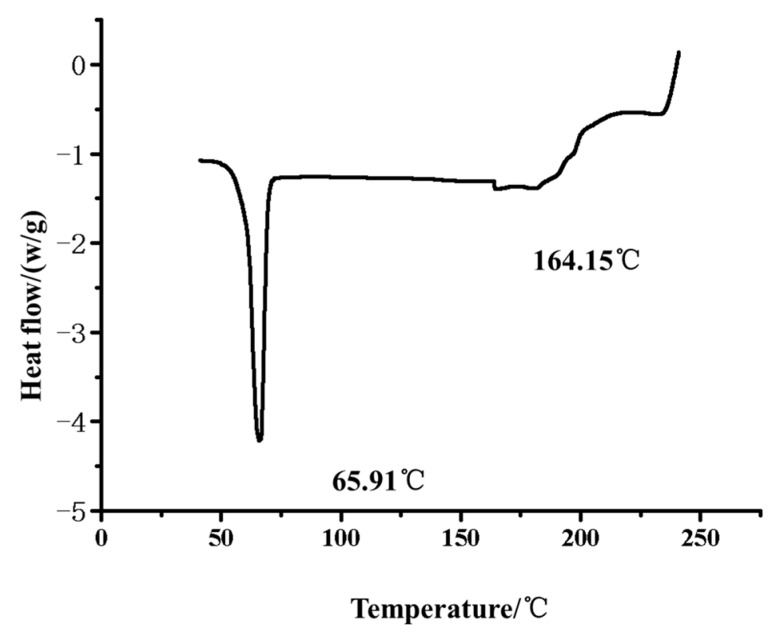
DSC curves of PSVNa.

It can be clearly seen that no obvious changes occurred before 250 °C in the TGA curve (Figure 3). It can be seen in the DSC curve (Figure 4) that the melting point of PSVNa is 65.9 °C, while the DSC curve began to fluctuate when the temperature was above 164 °C due to the exothermic reaction induced by double bond polymerization.

**Figure 5:**
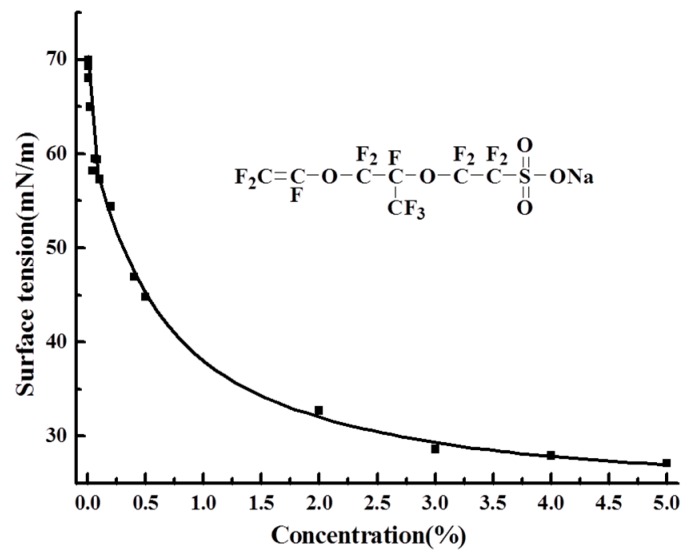
Curve of surface tension versus concentration of PSVNa.

Surface tension of PSVNa has been measured using interfacial tension meter and the results are shown in Figure 5. The surface tension decreases with increasing concentrations and the lowest surface tension is reached at 26 mN/m, where the CMC is 4.0%. The results show that the surface activity of PSVNa has no obvious advantages compared to other surfactants, which is due to the relatively short hydrophobic chain segment. However, when the emulsifier is bonded to the polymer chain, its hydrophobic chain segment will be greatly increased, and excellent surface properties can be expected.

### 3.2. Application of PSVNa as an Emulsifier in Emulsion Polymerization of DFHMA/MMA

The emulsion polymerization of fluorinated acrylate is easy to implement, and the obtained emulsion can be used as a coating and finishing agent. The application of PSVNa as an emulsifier in emulsion polymerization of DFHMA/MMA was investigated.

Comparison of emulsion polymerization using different emulsifiers is shown in Table 1. According to Table 1, the induction period of all emulsion polymerization is very short, and the conversion is relatively high. Because of the strong hydrophobicity of fluorinated monomers and polymers, they are usually difficult to emulsify and are prone to gel formation. Meanwhile, when using PSVNa or PSVNa/SDS as emulsifiers, the gel contents in the emulsions are very low, and the emulsions can be stored at room temperature for at least three years. The emulsions are believed to have excellent stability because the fluorocarbon polymerizable surfactants are covalently linked to the polymer chains, which has been verified in this paper.

**Figure 6:**
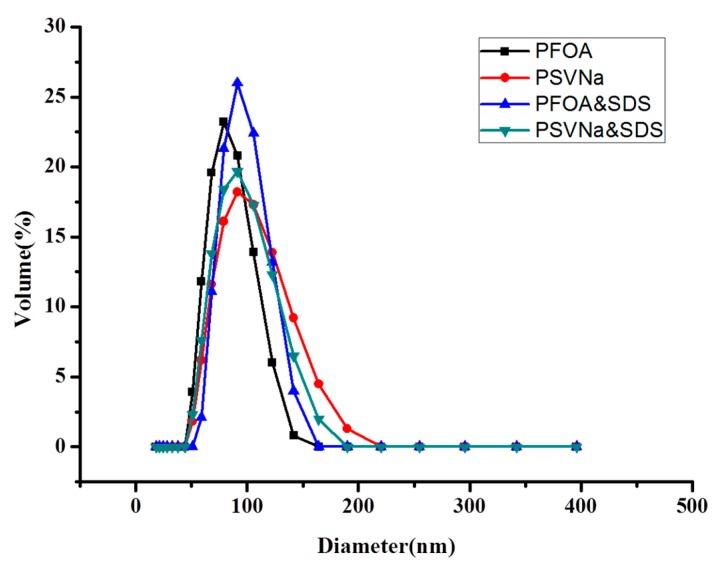
Particle size and distribution of fluorinated acrylate latex with different emulsifier.

According to Figure 6, the particle size of latex particles with different emulsifiers is less than 100 nm and the distribution is very narrow, which shows that emulsifiers have a good emulsifying effect.

**Figure 7:**
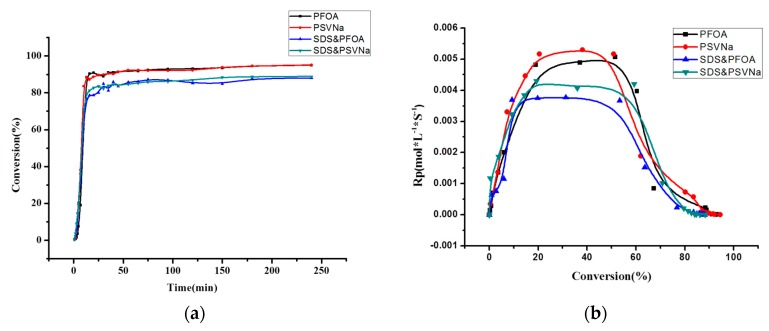
(**a**) Variations of conversion with time for different emulsifier. (**b**) Variation of *R*p with conversion for different emulsifier.

The kinetics of emulsion polymerization has also been investigated. Figure 7a shows that all emulsion polymerizations achieve high conversion rates in a very short period. In order to investigate the influence of emulsifier on the polymerization rate, the reaction rates of emulsion polymerization using different emulsifiers were calculated by the following equations:

*R*p = (M_0_) × dc/dt (2)

The calculated relationship between reaction rate (*R*p) and conversion of monomer was shown in Figure 7b. Three typical stages of emulsion polymerization, acceleration, constant rate, and deceleration periods can be observed. It can be found that *R*p at a constant rate period of emulsion polymerization using PSVNa as an emulsifier is higher than that using PFOA as an emulsifier. It is known that *R*p is proportional to the concentration of latex particles. Under the same emulsifier concentration, the higher *R*p means that the number of latex particles generated is greater, indicating that the emulsifying effect of PSVNa is better than that of PFOA.

### 3.3. Characterization of the Obtained Copolymer

**Figure 8:**
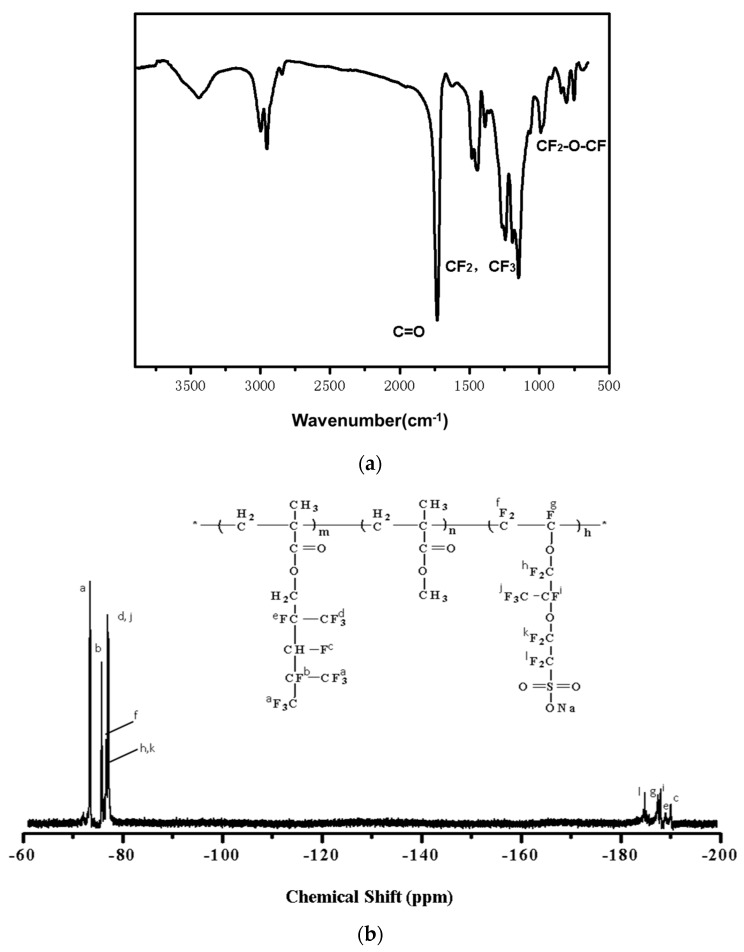
FTIR Spectra and ^19^F NMR spectrum of latex films.

The copolymer was obtained after it was demulsified and washed with methanol. Figure 8a is the FTIR spectra of synthesized polymer using PSVNa as an emulsifier. Besides the typical infrared vibration absorption peaks of C=O (1740 cm^−1^), CH_3_ and CH_2_ (1375, 1480, 2850–3000 cm^−1^), and CF_2_ and CF_3_ (1100–1300 cm^−1^), a peak at 985 cm^−1^ attributed to CF_2_–O–CF can also be observed, which proves that the emulsifier was covalently linked to the polymer chain successfully.

The ^19^F NMR spectrum of the obtained copolymer is shown in Figure 8b. There are no NMR signals of CF_2_=CF (−113, −121, −136 ppm), but some fluoride signals from the PSVNa segment can be observed.

Both FTIR and ^19^F NMR results clearly show that fluorocarbon polymerizable surfactants are indeed covalently linked to the polymer chains during emulsion polymerization.

### 3.4. Analysis of PSVNa Residue in Latex Films

The latex film was obtained by direct drying of emulsion. After extraction, the extraction liquid was diluted and detected by LC-MS. As reference samples, the standard solutions of PSVNa with different concentrations were also prepared and tested. Table 2 shows that, when the concentration of PSVNa is lower than 10 ppm, it cannot be detected by LC-MS. When the extraction was diluted to 1.0 L, the signal of PSVNa cannot be detected, which means that the concentration of PSVNa is less than 10 ppm. According to the content of the extracted PSVNa and the added PSVNa, it can be calculated that more than 95% of PSVNa has been covalently linked to the polymer chains during emulsion polymerization. The reduction or disappearance of small molecule fluorinated surfactants is favorable for environmental protection.

### 3.5. Immersion Behavior of the Latex Films

The surface properties of the obtained latex films were investigated (Table 3). The results show that the contact angles of the latex films will not decrease using PSVNa as an emulsifier.

## 4. Conclusions

In this study, a novel environmentally friendly fluorocarbon polymerizable surfactant, perfluoro (4–methyl–3, 6–dioxaoct–7–ene) sodium sulfonate (PSVNa), was synthesized and characterized. The results show that, when the concentration was above 4%, the lowest surface tension of PSVNa could reach 26 mN/m. Although it has no obvious advantages on surface tension compared with other fluorocarbon surfactants, the surfactants can be covalently linked to the polymer through double bonds at the beginning of polymerization, so its surface tension improved greatly because of the increase in its hydrophobic segment size. Furthermore, the emulsion polymerization of DFHMA/MMA using PSVNa and PSVNa/SDS as emulsifiers were investigated in terms of reaction kinetics, particle size, and stability of emulsion. The results indicated that the surfactant has excellent emulsifying properties and could be used as an alternative to PFOA.

## Figures and Tables

**Table 1:** Influence of emulsifier on emulsion polymerization.

Emulsifier			Induction time (min)	Conversion (wt %)	Gel content (wt %)	Storage stability (d)
SDS	PFOA	PSVNa				
0	—	2 wt %	1	92	0.6	≥1100
1 wt %	—	1 wt %	1	85	0.9	≥1100
1 wt %	1 wt %	—	2	88	3.6	≥180
0	2 wt %	—	2	86	5.1	≥180

**Table 2:** Residue detection results of PSVNa.

Sample	LC-MS results (PSVNa signal)
PSVNa Solution (50 ppm)	Yes
PSVNa Solution (10 ppm)	Yes
PSVNa Solution (5 ppm)	No
PSVNa Solution (1 ppm)	No
Extraction Liquid (diluted to 0.9 L)	Yes
Extraction Liquid (diluted to 1 L)	No

**Table 3:** Contact angle of latex films with different emulsifiers.

Emulsifier			DFHMA/MMA	Contact angel (°)
SDS	PFOA	PSVNa		
0	—	2 wt %	40/60	98
1 wt %	—	1 wt %	40/60	94
1 wt %	1 wt %	—	40/60	87
0	2 wt %	—	40/60	88
